# Supplementary material for: Chimeric Peptidoglycan Hydrolases Kill Staphylococcal Mastitis Isolates in Raw Milk and within Bovine Mammary Gland Epithelial Cells
Source: Viruses. 2022 Dec 15;14(12):2801. doi: 10.3390/v14122801 (PMC9781970; doi:10.3390/v14122801)
Supplement: Supplementary file 1 [file viruses-14-02801-s001.zip › viruses-2054197-supplementary.pdf]

SUPPLEMENTARY MATERIALS

Supplementary Table S1: Bacterial strains used in this study for protein expression (*E. coli*) and functional assays (*S. aureus*).

Species	Strain	Comments	Source[1]
S. aureus	Cowan	Clinical isolate (Septic arthritis), ATCC 12598	1
S. aureus	Newbould 305	Clinical isolate (Bovine mastitis), ATCC 29704	1
S. aureus	Mastidis	Clinical isolate (Bovine mastitis)	2
S. aureus	M2071	Clinical isolate (Bovine mastitis), Genotype R	3
S. aureus	M5512VL	Clinical isolate (Bovine mastitis), Genotype B	3
S. aureus	M5072	Clinical isolate (Bovine mastitis), Genotype B	3
S. aureus	USA300 JE2	Clinical isolate (SSTI, MRSA), NARSA NR-46543	4
E. coli	BL21-Gold(DE3)	Tet^R	5
E. coli	SURE®	Tet^R, Kan^R	6
E. coli	XL1-Blue MRF′	Tet^R	6

[1] Strains were originally obtained from: 1, ATCC, Manassas, USA; 2, Roger Stephan, University of Zurich, Zurich, CH; 3, Hans Ulrich Graber, Agroscope, Liebefeld, CH; 4, NARSA, Herndon, USA; 5, Agilent Technologies, Santa Clara, USA (Cat. No. 230132); 6, Stratagene, La Jolla, USA. Abbreviations: ATCC, American Type Culture Collection; Kan^R, Kanamycin resistance; MRSA, methicillin-resistant *Staphylococcus aureus*; NARSA, Network on Antimicrobial Resistance in *Staphylococcus aureus*; Tet^R, tetracycline resistance; SSTI, skin and soft-tissue infection.

Supplementary Table S2: Minimum bactericidal concentrations of PGHs against six *S. aureus* mastitis isolates and the MRSA strain USA300 in UHT milk. Bacteria were challenged with serially diluted PGHs in UHT milk and MBC was determined by plating after two hours. Average MBCs (± SEM) were calculated from biological triplicates.

	**MBC [nM] of selected peptidoglycan hydrolases**						
	**LST**	**ALE-1**	**M23LST(L)_ SH3b2638**	**(M23LST)2_ SH3bALE-1**	**CHAPK_ SH3bLST**	**CHAPGH15_ SH3bALE1**	**CHAPH5_ LST_H**
**Newbould**	23.4 ± 4.5	31.3 ± 0.0	93.8 ± 18.0	292.0 ± 41.7	167.0 ± 20.8	250.0 ± 0.0	1000.0 ± 0.0
**Mastidis**	7.8 ± 0.0	10.4 ± 2.6	15.6 ± 0.0	62.5 ± 0.0	20.8 ± 5.2	46.9 ± 9.0	229.0 ± 20.8
**M2071**	7.8 ± 0.0	10.4 ± 2.6	13.0 ± 0.0	104.0 ± 20.8	31.3 ± 0.0	62.5 ± 0.0	188 ± 36.1
**M5512VL**	6.5 ± 1.2	5.2 ± 1.3	9.1 ± 1.3	31.3 ± 0.0	26.0 ± 5.2	41.7 ± 10.4	62.5 ± 18.0
**M5702**	9.1 ± 3.5	10.4 ± 2.6	18.2 ± 6.9	141 ± 59.2	41.7 ± 10.4	93.8 ± 36.1	281.0 ± 118.0
**USA300**	11.7 ± 2.6	14.3 ± 1.3	28.6 ± 2.6	135.0 ± 27.6	104.0 ± 20.8	250.0 ± 0.0	333.0 ± 41.7

Supplementary Table S3: Minimum bactericidal concentrations of selected PGHs in raw milk against *S. aureus* Newbould 305. $2.5x10^5$ bacteria were challenged with serially diluted PGHs and MBC was determined after two hours by plating. Average MBCs (± SEM) were calculated from three (10% and 30% raw milk) and four (100% raw milk) biological replicates.

	**MBC [nM] of selected peptidoglycan hydrolases**						
	**LST**	**ALE-1**	**M23LST(L)_ SH3b2638**	**(M23LST)2_ SH3bALE-1**	**CHAPK_ SH3bLST**	**CHAPGH15_ SH3bALE1**	**CHAPH5_ LST_H**
**Raw milk (100%)**	15.6 ± 0.0	24.1 ± 7.6	31.3 ± 0.0	172.0 ± 46.9	2125.0 ± 657.0	1125.0 ± 239.0	1188.0 ± 373.0
**Raw milk (33%)**	2.1 ± 0.4	3.9 ± 0.0	78.1 ± 23.9	78.1 ± 23.9	1000.0 ± 0.0	583.0 ± 220.0	750.0 ± 250.0
**Raw milk (10%)**	0.5 ± 0.0	1.8 ± 0.3	2.3 ± 0.7	31.3 ± 4.5	1125.0 ± 473.0	750.0 ± 250.0	375.0 ± 125.0

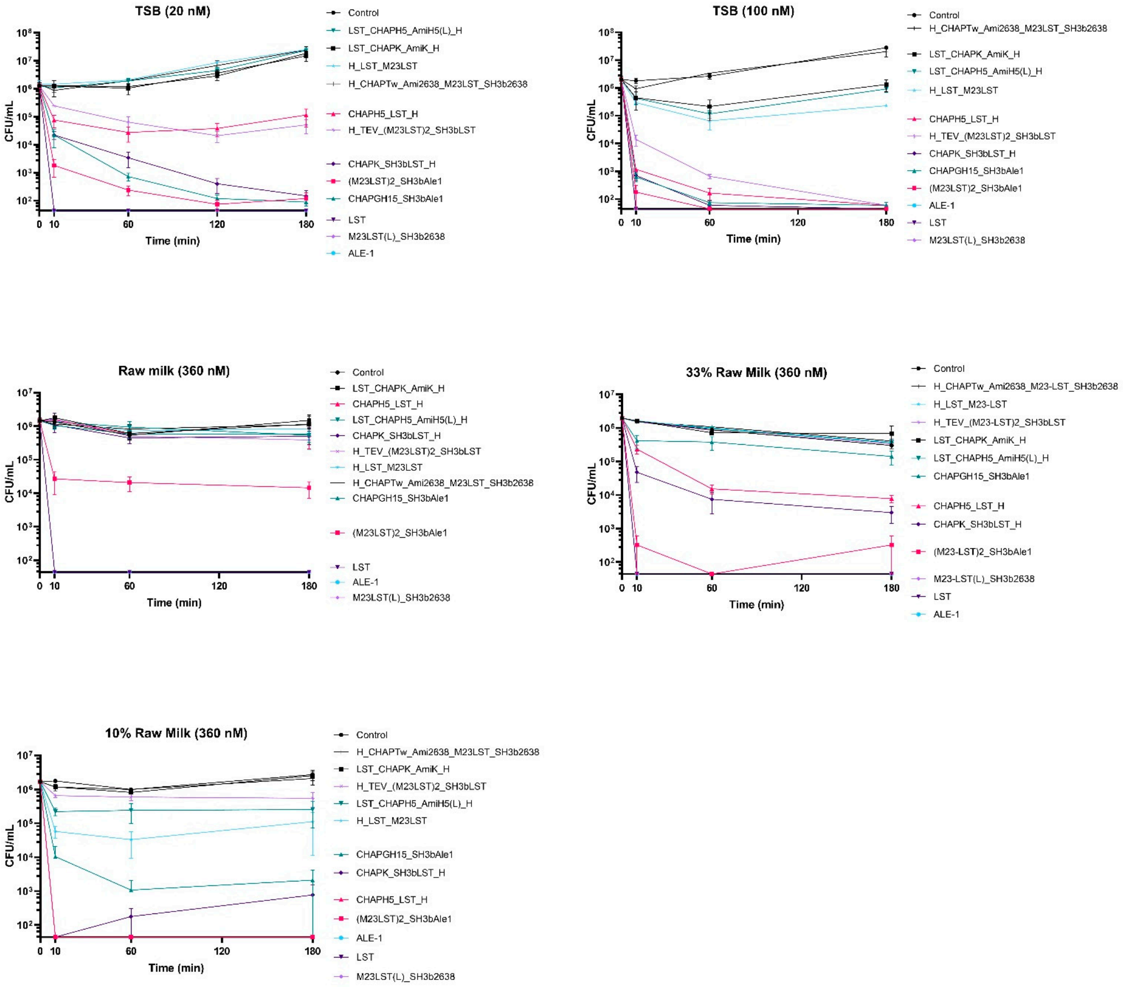

Supplementary Figure S1: Staphylolytic activity of PGHs in growth media and raw milk over time. *S. aureus* Newbould 305 (10^6 CFU/mL) was challenged with 12 PGHs selected for high activity in raw milk in a microwell plate screening. Bacterial counts were determined after 10, 60 and 180 minutes by plating, and the mean CFU/mL (± SEM) was calculated from biological triplicates. The y-axis was cut at the detection limit (44 CFU/mL). Legends of PGHs are grouped next to each graph according to the final CFU/mL count after 180 minutes to facilitate readability.

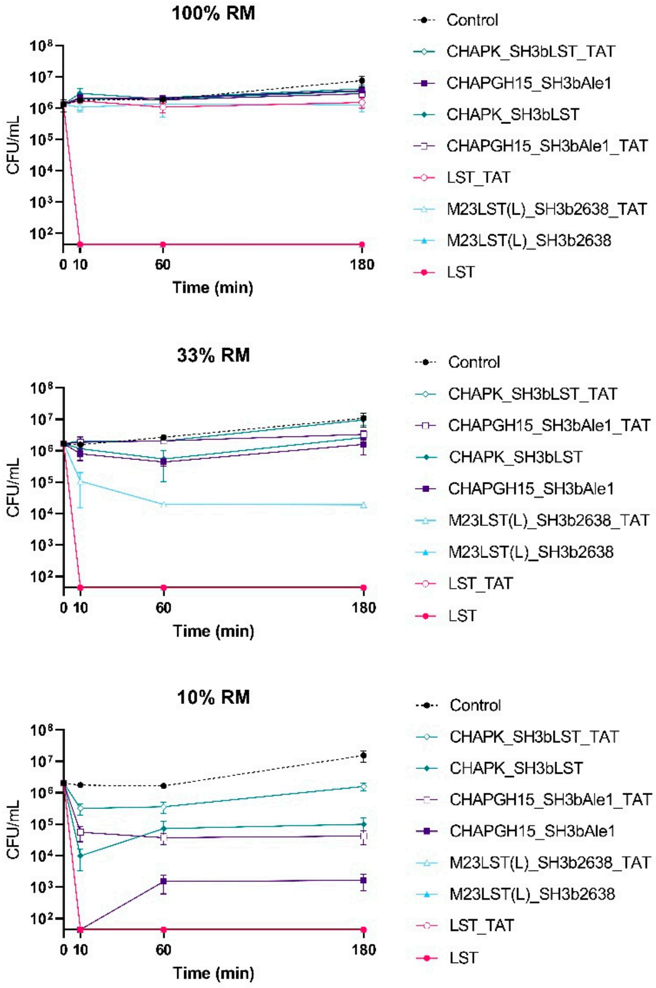

Supplementary Figure S2: Staphylolytic activity of four parental and CPP-fused PGHs as determined by time-kill assays. *S. aureus* Newbould 305 (10^6 CFU/mL) was treated with 360 nM enzyme in raw milk or milk diluted with DBPS. Bacterial counts were determined after 10, 60, and 180 minutes by plating. Plotted are the mean (± SEM) CFU/mL determined at each timepoint in biological triplicates. Y-axis was cut at the detection limit (44 CFU/mL). Legends on the right are sorted according to the activity of the enzyme after 180 minutes.
